# Leukocyte Surface Biomarkers for Clinical Diagnosis of Sporadic Alzheimer’s Disease

**DOI:** 10.1002/alz.095733

**Published:** 2025-01-09

**Authors:** Abe Durrant, Ben J Gu, Samuel P. Dickson, Jessie Nicodemus‐Johnson, Garrett B Duncan, Suzanne B. Hendrix

**Affiliations:** ^1^ Pentara Corporation, Salt Lake City, UT USA; ^2^ Innate Phagocytosis Laboratory, Jumar Bioincubator, Melbourne, VIC Australia

## Abstract

**Background:**

Mounting evidence indicates that the accumulation of amyloid in AD starts 20 to 30 years before clinically detectable cognitive impairment is observed, suggesting the presence of a long period of asymptomatic AD. Although the specific onset of this preclinical period is difficult to target, it is potentially significant to identify subjects in this asymptomatic preclinical stage of the disease. This is because newly developed disease modifying treatments may have increased effectiveness if begun during this asymptomatic period. Currently, the preclinical phase of AD is identified in asymptomatic individuals as abnormal amyloid levels detected via neuroimaging or fluid biomarkers. However, this approach is not feasible as a routine screening tool in a clinical environment, therefore there is a critical need to develop blood‐based, cost‐effective screening tools to detect AD in asymptomatic patients. Because of this, the search for preclinical blood‐based biomarkers has become a major focus.

**Method:**

200 Participants were randomly selected from the Australian Imaging, Biomarkers, and Lifestyle study (AIBL). A total of 34 leukocyte antigens were examined by flow cytometry immunophenotyping. Leukocyte markers were used in addition to age, ApoE4 status, years of education, and sex to predict the PET Aβ status. Data were analyzed by logistic regression and receiver operating characteristic (ROC) analyses.

**Result:**

We identified 12 specific leukocyte markers that were differentially expressed in cognitively normal (CN) patients designated as amyloid negative compared with CN individuals who are amyloid positive (Amyloid PET Centiloid >25). Combinations of these 12 markers produced AUC values as high as 0.94 in‐sample, and 0.90 out‐of‐sample. Specifically, markers CD11c, CD59, CD91, and CD163 had high performance when predicting amyloid positivity. These markers also showed strong predictive performance at other levels of Centiloid, maintaining AUCs greater than 0.85.

**Conclusion:**

Results suggest that leukocyte surface biomarkers involved in Aβ transportation, innate phagocytosis and completement mediated clearance pathways are the first blood‐based biomarkers that can accurately predict amyloid load and detect amyloid at 25 centiloids. These biomarkers could have a major impact on clinical practice by allowing primary care physicians to identify individuals at high risk of having amyloid burden in their brains with a simple blood test.

